# Structural and Mechanistic Evidence for Calcium Interacting Sites in the HIV Transmembrane Protein gp41 Involved in Membrane Fusion

**DOI:** 10.1021/acs.biochem.2c00372

**Published:** 2022-08-22

**Authors:** Yoel A. Klug, Roland Schwarzer, Thirupathi Ravula, Etai Rotem, Ayyalusamy Ramamoorthy, Yechiel Shai

**Affiliations:** † Department of Biomolecular Sciences, The Weizmann Institute of Science, Rehovot 7632701, Israel; ‡ Institute for Translational HIV Research, University Hospital Essen, University of Duisburg-Essen, Essen 45147, Germany; § Biophysics Program, Department of Chemistry, Macromolecular Science and Engineering, Biomedical Engineering, Michigan Neuroscience Institute, The University of Michigan, Ann Arbor, Michigan 48109-1055, United States

## Abstract

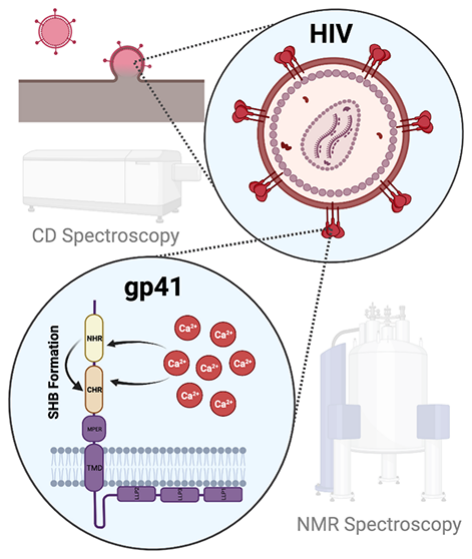

The HIV envelope protein gp160 comprises two subunits, gp120 and gp41, responsible for receptor binding and membrane fusion during viral entry, respectively. In the course of the membrane fusion process, gp41 undergoes a conformational change, leading to the formation of a six-helix bundle (SHB), which ultimately drives membrane fusion. The gp41 C-terminal and N-terminal heptad repeats (CHR and NHR) interact with one another to form the SHB, and this step can be targeted by peptide inhibitors, which are used in the clinic to mitigate HIV infection. Here, we discover the calcium interaction motifs (CIMs) in the gp41 CHR and NHR regions *via* NMR spectroscopy. We find that the assembly of the CHR–NHR SHB is facilitated in Ca^2+^-containing media and impaired in CIM mutants. Of note, the clinically approved, gp41-derived fusion inhibitor T20, which does not contain the CIM motif, exhibits reduced inhibitory efficiency when challenged with calcium. This finding could have important implications for the development of better fusion inhibitors for HIV.

## Introduction

One of the critical steps in HIV infection is the fusion of the viral membrane with its CD4^+^ host cells’ membrane.^1^ This process is mediated by the two noncovalently bound envelope glycoprotein subunits gp120 and gp41, which bind host cell receptors and mediate membrane fusion, respectively.^2−4^ Binding of gp120 to CD4, followed by binding to one of the two coreceptors CXCR4 or CCR5, induces a conformational change that exposes the fusion-mediating subunit, gp41.^5,6^ This subunit is composed of the fusion peptide (FP), N-terminal heptad repeat (NHR), loop, C-terminal heptad repeat (CHR), transmembrane domain (TMD), and a cytoplasmic tail.^7^ During the fusion process, the NHRs are packed into the CHR to form the six-helix bundle (SHB), bringing the viral and cellular membranes to close proximity, ultimately promoting fusion.^2,4,8^

A number of previous publications have reported an involvement of Ca^2+^ ions in HIV entry and membrane fusion. The Blumenthal group was likely the first to describe that Env-mediated membrane fusion is impaired in the absence of Ca^2+^, whereas receptor binding was found to be unaffected.^9^ Additional data support the notion that calcium plays a role in HIV infection, possibly in the fusion process involving SHB formation.^9−13^ However, to date, no direct evidence has been provided showing the mechanistic role of calcium in this process.

**Figure 1:**
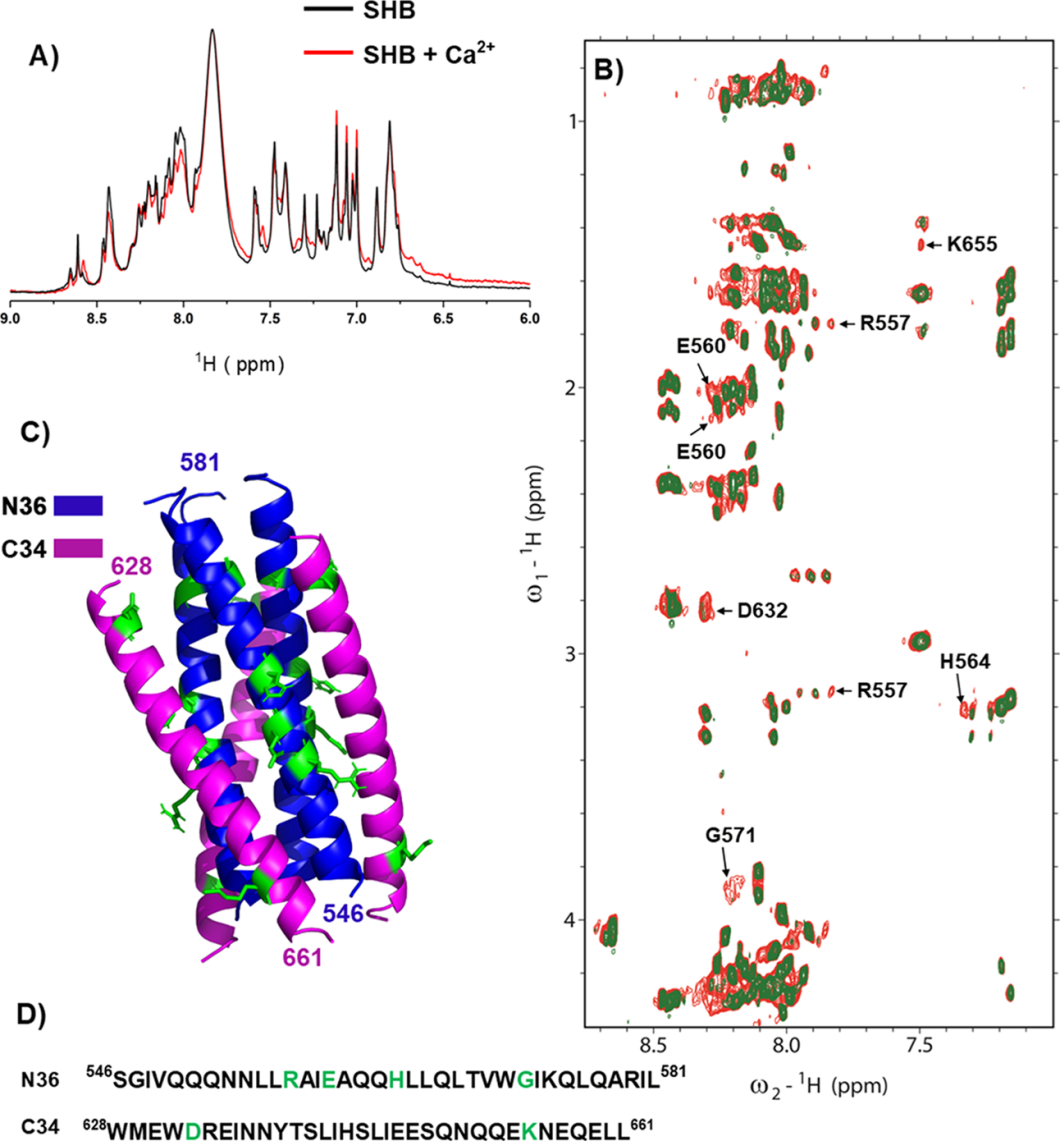
Interaction sites of Ca^2+^ ions on the SHB revealed by NMR spectroscopy. (A) Selected amide region of the ^1^H NMR spectrum of SHB (200 μm) in Tris-D11 buffer (5 mM, pH 7.4) recorded at 308 K, without Ca^2+^ (blue) and with 5 mM Ca^2+^ (red). (B) 2D ^1^H/^1^H TOCSY NMR spectrum of SHB (200 μm) in Tris-D11 buffer (5 mM, pH 7.4) recorded at 308 K. Resonances were tentatively assigned using the chemical shift values reported in the literature.^15^ (C) X-ray crystal structure reported for SHB (PDB: 1aik). N36—blue and C34—purple. The residues shown in green showed a decrease in intensity in the TOCSY spectrum. (D) Amino acid sequence and residue numbering used for the resonance assignment in the 2D TOCSY NMR spectrum.

In this study, we aimed to specifically test the role of calcium in HIV fusion, specifically SHB formation. Using nuclear magnetic resonance (NMR) spectroscopy, we have identified amino acids within the CHR and NHR that interact with Ca^2+^ ions (Figure 1). Based on these observations, we generated several peptides mutated in these specific amino acids (Table 1). To evaluate the effect of Ca^2+^ on SHB formation, we evaluated these peptides for their ability to form SHB *in vitro* using circular dichroism (CD), as well as *in cellulo*, by comparing their ability to inhibit HIV cell–cell fusion in the presence or absence of Ca^2+^ and Mg^2+^ as a control. We found that Ca^2+^ promoted SHB formation between the wildtype (WT) CHR (C34) and NHR (N36) motifs both *in vitro* and *in cell culture*. A mutation in C34 enhanced its SHB oligomerization and cell–cell fusion at high Ca^2+^ levels, whereas N36 mutations were either not affected or abrogated in their ability to form a SHB *in vitro* and *in cellulo*. Mg^2+^ had no effect, suggesting a Ca^2+^-specific interaction. Lastly, we compared C34 to the clinically used T20 (Enfuvirtide). T20 is a gp41-derived peptide that shares a large portion of its sequence with C34 but only partially overlaps with the calcium-binding site (Table 1). We show that Ca^2+^ enhances C34’s ability to form an SHB and inhibit cell–cell fusion as opposed to T20. In light of our findings, we propose a specific Ca^2+^ binding site comprising both N36 and C34. Overall, our data suggests that the effect of calcium on HIV fusion is mediated through the interaction of calcium with specific residues within the SHB to support its formation and stabilization.

**Table 1:** Sequences of Peptides Used in This Study

peptide designation	sequence^a^
CHR Peptides	
**C34**	WMEWDREINNYTSLIHSLIEESQNQQEKNEQELL
**C34D-A**	WMEW**A**REINNYTSLIHSLIEESQNQQEKNEQELL
**T20**	YTSLIHSLIEESQNQQEKNEQELLELDKWASLWNWF
NHR Peptides	
**N36**	SGIVQQQNNLLRAIEAQQHLLQLTVWGIKQLQARIL
**N36G-A**	SGIVQQQNNLLRAIEAQQHLLQLTVW**A**IKQLQARIL
**N36 3A**	SGIVQQQNNLL**A**AIAAQQ**A**LLQLTVW**G**IKQLQARIL

a All sequences are derived from the HXB2 strain of HIV-1.

## Materials and Methods

### Peptide Synthesis

Peptides were synthesized on Rink Amide MBHA resin by using the F-moc strategy as previously described^14^ on a Liberty Blue peptide synthesizer CEM. All peptides were cleaved from the resin by a trifluoric acid, ddw, and TFA/DDW/TES [93.1:4.9:2 (v/v)] mixture, and purified by reverse-phase high-performance liquid chromatography (RP-HPLC) to >95% homogeneity. The molecular weight of the peptides was confirmed by platform LCA electrospray mass spectrometry. The day before administration of peptides into biological assays, peptides were dissolved in 95% TFA that was then evaporated by N_2_. Next, peptides were dissolved in 50% acetonitrile and lyophilized overnight. Before administration of peptides into *in vitro* and *in cellulo* cell culture experiments, peptides were dissolved in DMSO. Cells were cultured in a medium not exceeding 0.5% DMSO concentrations.

### NMR Experiments

All NMR experiments were performed using 200 μM peptide. 10 mM Tris-D11, pH 7.4 buffer was used. 10% D_2_O was added for locking in NMR experiments. 1D ^1^H NMR spectra were recorded at 308 K on a Bruker AVANCE 900-MHz NMR spectrometer equipped with a cryoprobe. 64 scans were acquired, and 3 Hz line broadening was used for processing the data. 2D total correlated spectroscopy (TOCSY) NMR spectra were acquired using the following parameters: 80 ms mixing time, 1 s recycle delay, 16 number of scans, and 512 t1 points. A spectral width of 12 ppm was used. All spectra were processed using Bruker Topspin 2.0 (Bruker Co., Billerica, MA). The TOCSY spectra were analyzed using Sparky.

### Circular Dichroism

CD measurements were performed by using an Applied Photo physics spectropolarimeter. The spectra were scanned using a thermostatic quartz cuvette with a path length of 1 mm. Wavelength scans were performed at 25 °C; the average recording time was 7 s, in 1 nm steps, in the wavelength range of 190–260 nm and recordings were done in triplicates. Each peptide concentration of 25 μM was tested in a PBS without calcium or magnesium or with the addition of calcium or magnesium as detailed for each experiment.

### Cell–Cell Fusion

Effector cells were the Env expressing cells HL2-3, a HeLa-derived cell line which constitutively expresses the HXB2 strain of the HIV-1 Env glycoprotein along the Tat protein, and as target cells, TZM-bl cells were used. The fusion of HL2-3 cells with TZM-bl cells was assessed through luciferase expression. The TZM-bl cells were seeded at 2 × 10^4^ cells/well overnight in 96-well plates. The medium was then aspirated from each well and replaced with serum-free DMEM containing 40 μg/mL DEAE-dextran. Stock dilutions of each peptide were prepared in DMSO so that each final concentration was achieved with 1% dilution. Upon addition of the peptides, the HL2-3 cells were added to the TZM-bl cells in serum-free DMEM containing 40 μg/mL DEAE-dextran at a 1:1 cell ratio. The cells were cocultured at 37 °C for 6 h to allow the fusion to occur. Luciferase activity was analyzed using the Steady-Glo luciferase assay kit (Promega). Fitting of the data points was performed according to the following equation, derived from Hills’ equation
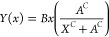


In brief, in this equation, *B* is the maximum value; therefore, it equals 100% fusion, *A* is the value of an inhibitory concentration at 50% viral infectivity (IC_50_), and *c* represents Hill’s coefficient. For the fitting, we uploaded the *X* and *Y* values of the data into a nonlinear least-square regression (curve fitter) program that provided the IC50 value (parameter A).

### Statistical Analysis

A one-tailed Student’s *T*-test was used. *P* < 0.05 was considered significant. Analyses were done using GraphPad Prism (data analysis software) version 6.05 (*P ≤ 0.05, **P ≤ 0.01, and ***P ≤ 0.001). Results are displayed as mean ± SEM.

## Results

### Calcium-Binding Sites on the SHB Revealed by NMR

To study the effect of calcium ions (Ca^2+^) on SHB, we performed 1D ^1^H NMR experiments in the presence of Ca^2+^ (Figure 1A). Changes in the intensity were observed for several peaks, suggesting an effect of Ca^2+^. To analyze the residues affected by Ca^2+^, we performed a 2D ^1^H–^1^H TOCSY NMR experiment (Figure 1B). The TOCSY spectrum was tentatively assigned using the reported resonance assignment for SHB in the literature.^15^ It is worth noting that the chemical shift values obtained from the literature, used as a secondary reference for assignment purposes, were based on experiments performed at pH 6.0, whereas the experiments performed in this study were at pH 7.4. NMR spectra reveal that residues R557, E570, H564, and G571 from the N36 peptide and D632 and K655 from C34 exhibit reduced peak intensity. These observed changes in the NMR spectra indicate that these residues are likely to interact with Ca^2+^. To test the specificity of Ca^2+^ interaction, NMR experiments were also performed in the presence of Mg^2+^ (Figure S1). In the presence of Mg^2+^, the SHB structure was severely compromised, suggesting structural instability. Nevertheless, this shows that the effect observed with Ca^2+^ is specific and not due to the presence of a bivalent ion.

### Mutation of Predicted Calcium-Binding Site Results in Impaired SHB Formation *In Vitro*

**Figure 2:**
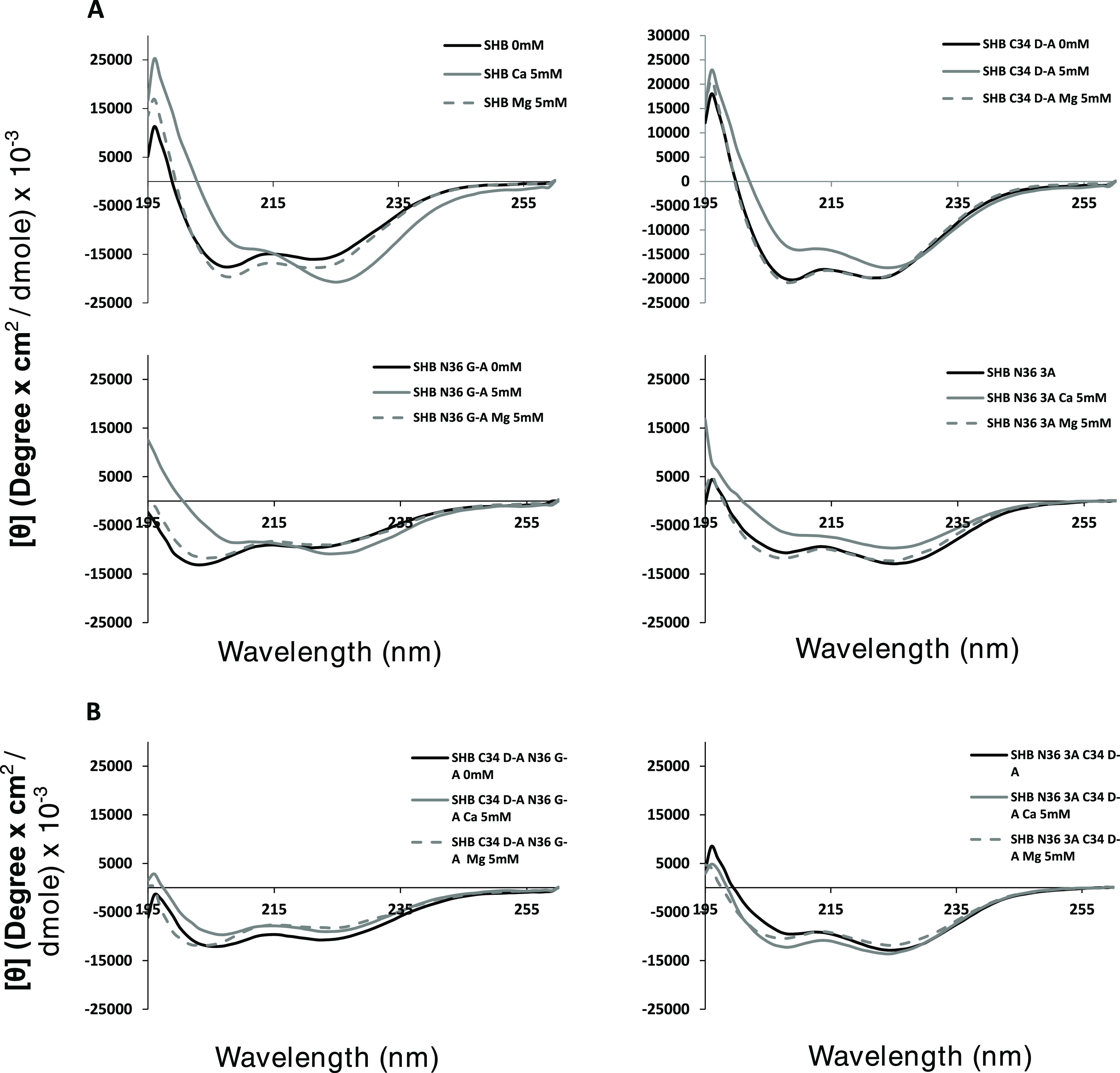
CD spectra reveal that Ca^2+^ but not Mg^2+^ affects SHB assembly through the residues detected by NMR. Peptides were measured at 25 μM in either PBS–/– (black) or PBS–/– supplemented with 5 mM calcium (gray) or magnesium (dashed gray) ions. (A) CD spectra WT SHB (SHB), and either WT N36 or C34 with a mutant corresponding to the SHB component. (B) CD spectra of SHB comprising mutant C34 and N36 peptides.

The NMR results pinpointed areas of interest for Ca^2+^ binding on the SHB: (i) D682 in the CHR and G622 in the NHR that sit across from one another; (ii) R607, E610, and H614 that sit in the center of the NHR and protrude outward away from the NHR trimer (Figure 1C). Accordingly, we synthesized three peptides in total with these residues swapped into alanine to preserve alpha helicity (Table 1): C34D-A, N46G-A, and N363A, where all the three central residues were mutated at once. We then assessed the ability of these peptides to form a SHB *via* CD spectroscopy (Figure 2). Initially, each peptide was mixed with a WT counterpart peptide in PBS–/– and PBS–/– with either 5 mM Ca^2+^ or 5 mM Mg^2+^. As a control, WT C34 and N36 were used (Figure 2A). As suspected, the WT SHB spectra shifted in Ca^2+^. Moreover, there was a pronounced dip at θ220, suggesting a more oligomeric state, as previously shown for coiled coils.^16−18^ Mg^2+^ had very little effect. Surprisingly, the three alanine mutants also responded in a similar fashion, with N36 G-A also becoming more alpha-helical.

Next, we mixed the C34 D-A mutant with either of the two N36 mutants as described above and analyzed their CD spectra. When C34 and N36 mutant peptides were used, Ca^2+^ did not have an effect on SHB formation when compared to PBS–/– and 5 mM Mg^2+^ (Figure 2B). This suggests that Ca^2+^ affects the interaction between the CHR and NHR rather than priming one component. The addition of Ca^2+^ did not affect the secondary structure of the peptides themselves (Figure S2), showing that the shift in secondary structure occurs during SHB formation and does not stem from an induced change in the peptide’s intrinsic secondary structure. We further scrutinized the interaction by comparing theoretical spectra of the WT and mutant SHBs to the experimental ones obtained in this study with and without 5 mM Ca^2+^. The theoretical spectrum portrays the additive spectra of the two peptides, which are input manually based on the spectra of each peptide alone and portrays the theoretical situation of both peptides in solution without interacting. The experimental spectra are obtained by measuring the peptides when mixed together. Hence, a shift between the theoretical and experimental spectra suggests a bona fide interaction and not one that results from both peptides just being in the same solution. All but one of the SHB derivatives tested in this study showed a bona fide interaction (Figure S3). The only exception was the SHB comprising WT C34 and N36 G-A without Ca^2+^, which showed no shift between theoretical and experimental spectra, suggesting no SHB formation. Since SHB formation was observed with N36 G-A 5 mM Ca^2+^, it is plausible that Ca^2+^ can drive the SHB formation of this mutant despite the mutation.

### Gp4- Mediated Cell–Cell Fusion Is Impaired When the Calcium-Binding Site Is Mutated

**Figure 3:**
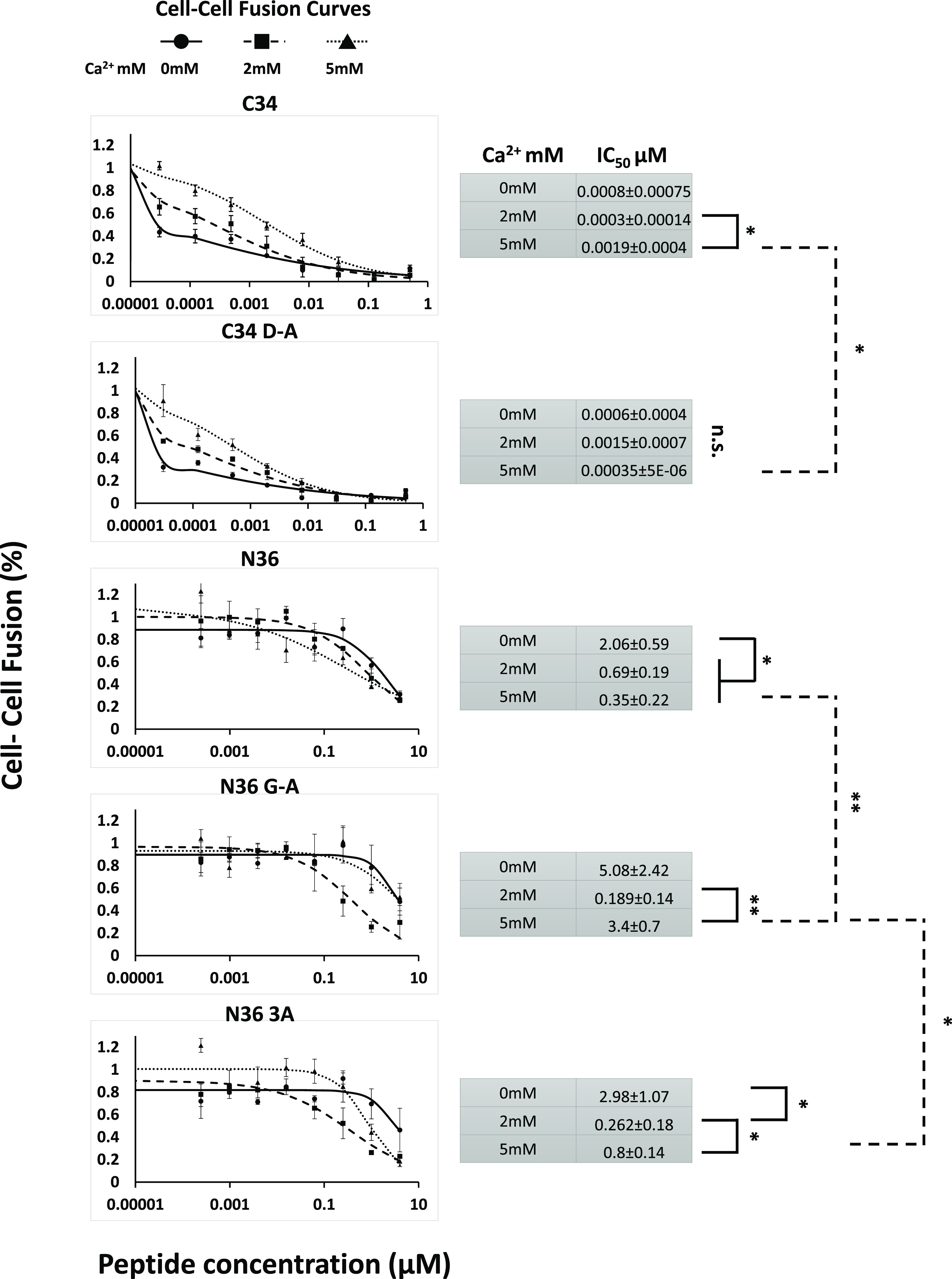
*In cellulo* evidence for the role of Ca^2+^ in SHB formation as seen in the cell–cell fusion assay. Cell–cell fusion assay utilizing TZM-bl as target cells and HL2/3 as effector cells. Dose-dependent cell–cell fusion. Apart from C34 D-A, calcium affected all the other peptide’s ability to inhibit cell–cell fusion. Left—shown curves and points averaged from three experiments. Right—IC_50_ average of peptides. *n* = 3. *P < 0.05 and **P < 0.01. Error bars represent ± S.E.M.

Following the *in vitro* experiments, we wanted to assess the impact of Ca^2+^ on membrane fusion *in cellulo*. NHR- and CHR-derived peptides act as competitors for SHB assembly. Therefore, their ability to bind counterpart SHB components can be assessed by their inhibitory effect on gp41 mediated cell–cell fusion, as previously described.^19−21^ Jernigan and co-workers have shown that calcium can enhance viral cell fusion of both HIV-1 and HIV-2.^13^ Therefore, we initially tested the effect of Ca^2+^ on an Env-dependent cell–cell fusion system. As expected, the addition of Ca^2+^ significantly increased the amount of cell–cell fusion observed when compared to media lacking Ca^2+^. A slight but nonsignificant effect was seen when Ca^2+^ levels were raised from 2 to 5 mM, suggesting saturation of the system (Figure S4). Next, we assessed the effect of the WT and mutant SHB-derived peptides on cell–cell fusion in the absence of calcium and with the addition of either 2 mM or 5 mM calcium (Figure 3). Both CHR-derived peptides showed strong inhibition of cell fusion with and without calcium. WT C34’s inhibitory ability was reduced slightly in 5 mM calcium as opposed to C34D-A that showed no change between calcium levels. In contrast to the CHR-derived peptides, all N36-derived peptides were strongly affected by the addition of calcium and showed a marked reduction in IC50. N36 mutant peptides exhibited an elevated IC50 at 5 mM calcium in contrast to WT N36 that retained its potency. Altogether, these findings suggest that calcium affects SHB formation *in cellulo*.

### T20-Based SHB Formation Is Not Influenced by Calcium

**Figure 4:**
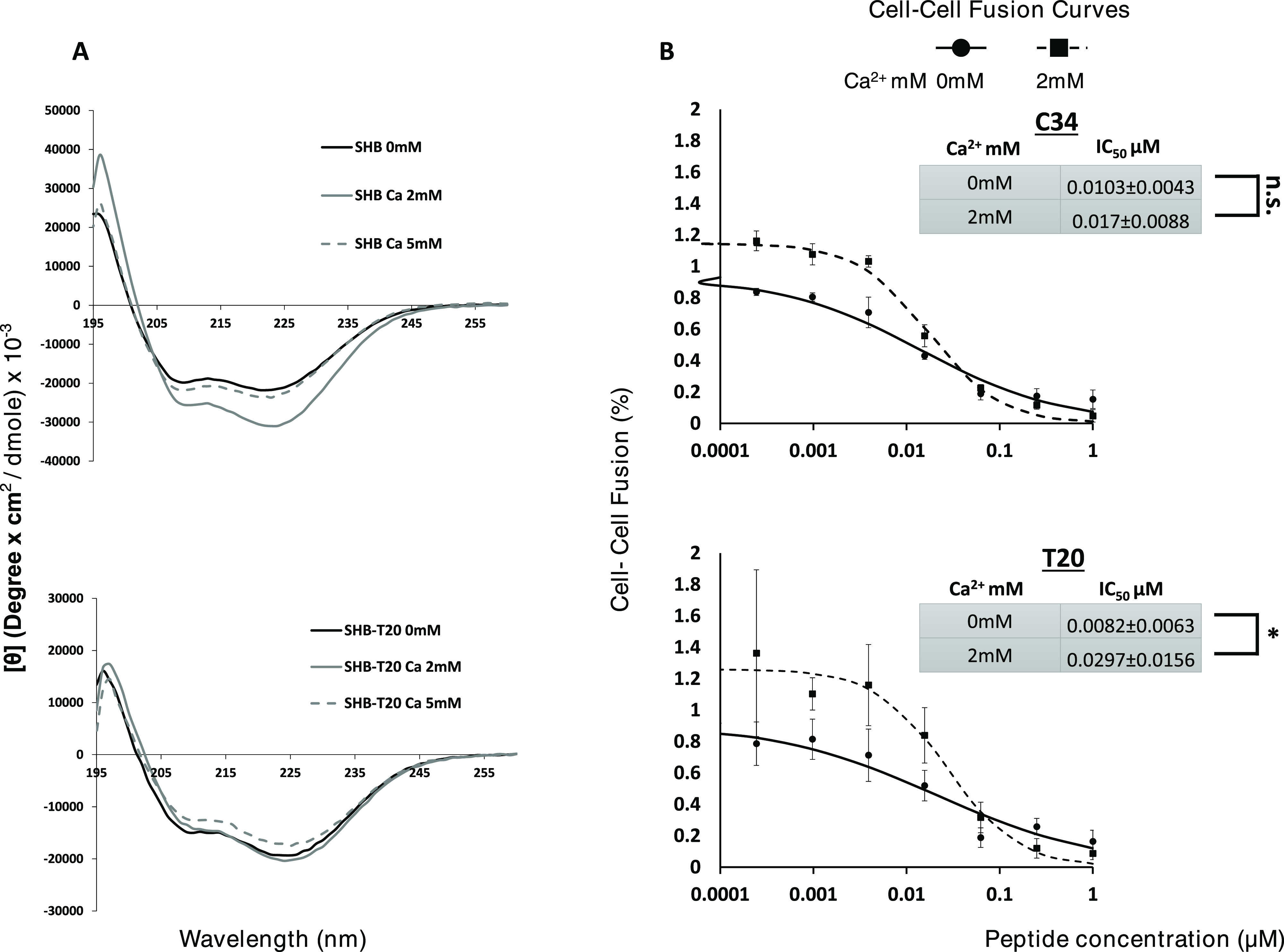
T20-based SHB formation does not respond to Ca^2+^*in vitro* and *in cellulo*. (A) CD spectra of SHB comprised N36 and either C34 (SHB) or T20 (SHB-T20) peptides. Peptides were measured at 25 μM in either PBS–/– (Black) or PBS–/– supplemented with either 2 mM (gray) or 5 mM (dashed gray) calcium. (B) Cell–cell fusion assay utilizing TZM-bl as target cells and HL2/3 as effector cells. Dose-dependent cell–cell fusion of C34 and T20 with shown calcium levels. Shown curves and points averaged from three experiments. *n* = 3. *P < 0.05. Error bars represent ± S.E.M.

Since SHB formation is crucial for successful HIV infection, it has emerged as a common drug target. One such clinically used drug that targets SHB assembly is the CHR-derived T20 (Enfuvirtide).^15,22^ Although derived from the CHR, T20 does not incorporate the calcium interacting residue found on C34 that was identified in this study (Figure 1 and Table 1). Therefore, we decided to test the influence of calcium on T20-based SHB formation. We first tested the ability of T20 and N36 to form an SHB (annotated SHB-T20) with and without the presence of calcium (Figure 4A) and magnesium (Figure S5) *via* CD spectroscopy. As a control, we used C34 and N36 (annotated SHB) (Figure 4A). In contrast to C34, T20 did not respond to the addition of calcium. Neither C34- nor T20-derived SHBs responded to the addition of magnesium (Figure S5). We further tested this *in cellulo via* cell–cell fusion assay as described for Figure 3. Both peptides exhibited a low IC50 without and with the addition of 2 mM calcium. However, in the presence of calcium, the IC50 of T20 rose significantly to over 3.5 the level exhibited without calcium. In contrast, C34 showed a minor nonsignificant increase. It is likely, that as T20 lacks the calcium interacting residue, its effectiveness as a competitor was reduced *versus* the WT CHR.

## Discussion

Enveloped viruses encapsulated their genome and proteins in lipid bilayers, derived from host cell membranes. A key step during their viral entry is the initial binding of the cellular surface receptor, followed by fusion between the virus envelope and target cell membranes. The entry of HIV is exclusively mediated by the trimeric HIV envelope protein (Env), consisting of the surface subunit gp120 and the transmembrane protein gp41. Env is a prototypical class I viral membrane fusion protein,^23,24^ and the structural mechanics as well as the molecular dynamics of the Env-mediated membrane fusion are today well understood. Before exposure to cellular receptors, Env exists in a native state on the surface of the virus or infected cells. HIV membrane fusion is initiated by conformational rearrangements in Env that are triggered by binding of gp120 to the receptor, CD4, and a coreceptor, CCR5 or CXCR4.^25^ This allows gp41 then to insert its amino-terminal FP domain into the cell membrane, forming a transient pre-hairpin intermediate that exposes two key helical regions (NHR and CHR).^26^ Subsequently, in its fusogenic conformation, gp41 positions the N and C helices in an antiparallel manner, called trimer-of-hairpins, the formation of which is mediated by intramolecular interactions between CHR and NHR. These interactions ultimately facilitate the juxtaposition of the virus and cell membranes, leading to membrane fusion and successful virus entry. The pre-hairpin intermediate is highly vulnerable to therapeutic intervention by inhibitory peptides mimicking heptad repeat regions. Such peptides block the intramolecular binding sites in a dominant negative manner, thus effectively halting the ensuing membrane fusion process.^26^

In this study, we have discovered Ca^2+^-responsive motifs in the gp41 CHR and NHR (termed CIMs), providing structural evidence from NMR spectroscopy for direct interactions between both heptad repeat regions and Ca^2+^ ions. CD revealed that SHB formation is Ca^2+^-sensitive and that mutations in the putative Ca^2+^ binding sites affect CHR–NHR interactions. In addition, a CHR-derived peptide, resembling the FDA-approved HIV fusion inhibitor T20, which lacks a CIM, exhibited a higher IC50 when applied to the cell–cell fusion assay in calcium-containing media.

Over the past 2 decades, several studies have established a link between the HIV fusion process and calcium ions. It was shown that fusion can be blocked by Ca^2+^ chelators, yet, without affecting the binding of gp120 to CD4, suggesting that calcium is directly involved in fusion.^9^ Following studies have identified a possible calcium-binding site within the extracellular part of gp41 that resembles the calcium-binding EF-hand structure, indicating that gp41 is a calcium-binding protein.^10^ Interestingly, HIV-1 infection of peripheral blood mononuclear cells has been suggested to be calcium-dependent,^11^ and some evidence indicates a distinct effect of Ca^2+^ on different types of HIV, as calcium ions have been shown to be crucial for HIV-1 but not for HIV-2 fusion.^13^ Yet, although Ca^2+^ is not required for HIV-2 fusion, the process is enhanced in the presence of Ca^2+^.^13^ Moreover, a recent study has demonstrated that the formation of the prefusion Env-CD4-coreceptor complexes triggers cell surface exposure of phosphatidylserine (PS) that is mediated through Ca^2+^ signaling, further demonstrating the role Ca^2+^ ions play in the HIV fusion process.^12^

Numerous studies have attempted to generate HIV-1 fusion inhibitors based on peptides derived from different regions of gp41^27−29^ Among the most studied peptides in this context is the clinically used T20 (Enfuvirtide)^22,30^ that blocks the formation of the SHB by binding the NHR region of gp41. A peptide encompassing the putative calcium-binding site in gp41 was shown to be a potent fusion inhibitor, yet, mutations in the calcium-binding site within this peptide or deletion of this site results in a lack of antiviral activity.^31^ The same peptide was shown to be dependent on calcium to bind the HIV mucosal receptor galactosyl ceramide.

Noteworthily, there is very limited evidence for a Ca^2+^ dependency of viral entry from other virus species. Dube and co-workers have recently reported Ca^2+^ involvement in the membrane fusion and entry of the Rubella virus from the family of Matonaviridae.^32^ The Rubella virus envelope protein E1 is a class II fusion protein and it was demonstrated that Ca^2+^, but not other bivalent cations, shape interactions of the protein’s membrane penetrating FPs. Interestingly, a similar Ca^2+^ dependency was reported for the FP of SARS CoV and SARS CoV-2 spike protein.^33,34^ Importantly, Straus *et al.* were able to repurpose pharmacological calcium channel blockers, which showed promise as a novel class of antivirals inhibiting SARS CoV-2 entry and spread.^33^ This result demonstrates that Ca^2+^-dependent entry can serve as a therapeutic target and could be exploited for the development of effective pan-viral drugs with beneficial pharmacological properties.

## Supporting Information Available

Figure S1: ^1^H NMR spectra of SHB in the presence of Mg^2+^. Figure S2: Figure showing that Ca^2+^ does not affect the overall secondary structure of WT and mutant C34 and N46 peptides. Figure S3: Theoretical and experimental spectra of SHB variations tested in this study. Figure S4: Calcium dependency of gp41-mediated cell–cell fusion. Figure S5: Figure showing that Mg^2+^ does not affect the structure of C34- and T20-derived SHBs (PDF)
