# Supplementary material for: Structural and Mechanistic Evidence for Calcium Interacting Sites in the HIV Transmembrane Protein gp41 Involved in Membrane Fusion
Source: Biochemistry. 2022 Aug 22;61(17):1915–22. doi: 10.1021/acs.biochem.2c00372 (PMC9454089; doi:10.1021/acs.biochem.2c00372)
Supplement: Supplementary file 1 — bi2c00372_si_001.pdf [file bi2c00372_si_001.pdf]

Supplementary results

Structural and mechanistic evidence for calcium interacting sites in the HIV transmembrane protein gp41 involved in membrane fusion.

Yoel A. Klug[1,2#], Roland Schwarzer[1,3*#], Thirupathi Ravula[4,5], Etai Rotem[1], Ayyalusamy Ramamoorthy[4], Yechiel Shai[1,*]

1 Department of Biomolecular Sciences, The Weizmann Institute of Science, Rehovot, 7632701, Israel

2 Current address: Sir William Dunn School of Pathology, University of Oxford, Oxford, OX1 3RE, UK

3 Institute for Translational HIV Research, University Hospital Essen, University of Duisburg-Essen, 45122, Germany

4 Biophysics Program, Department of Chemistry, Macromolecular Science and Engineering, Biomedical Engineering, Michigan Neuroscience Institute, The University of Michigan, Ann Arbor, MI 48109-1055, USA

5 Current address: The National Magnetic Resonance Facility at Madison (NMRFAM), Department of Biochemistry, University of Wisconsin-Madison, Madison, WI 53706

# equal contribution

* Corresponding author

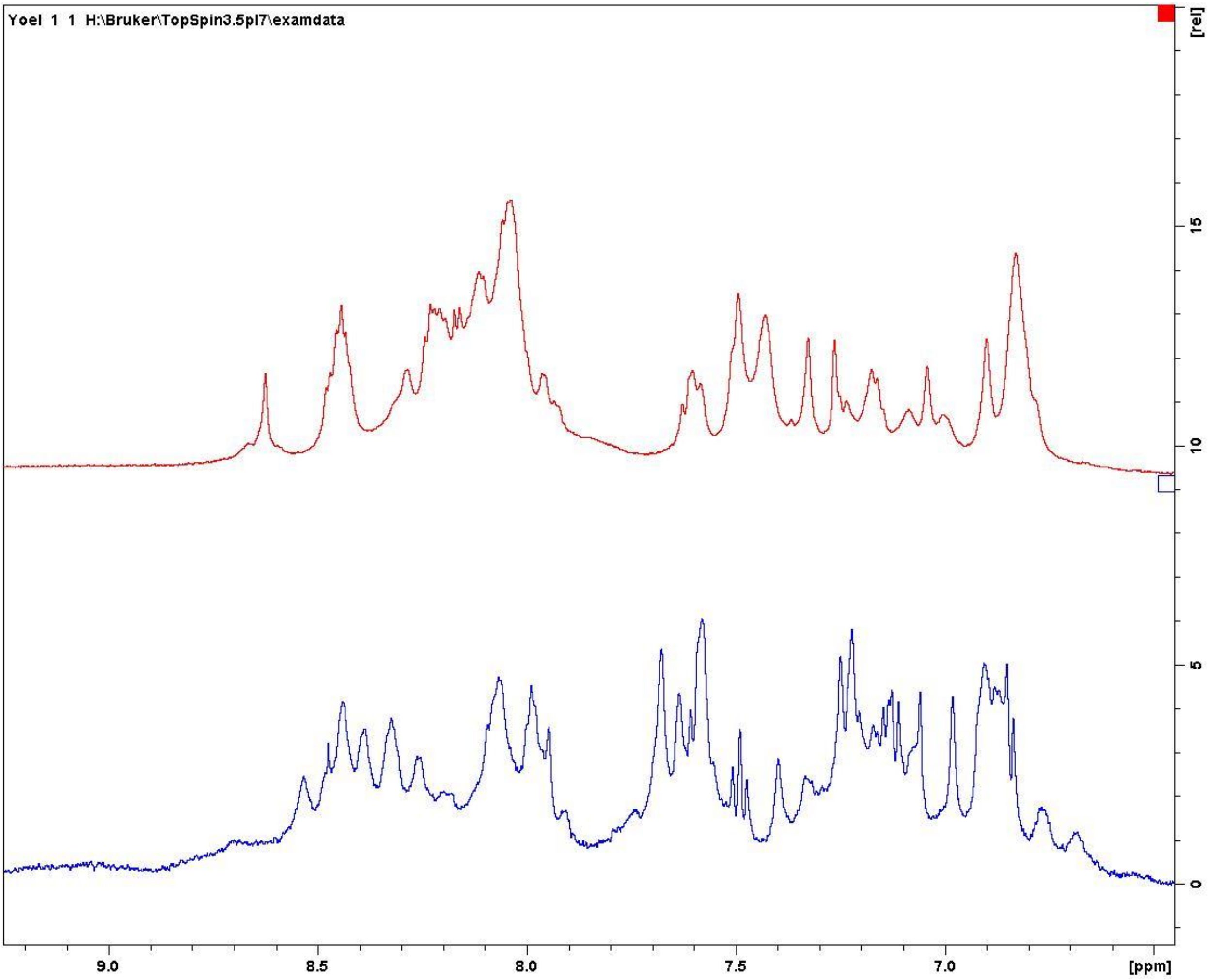

Figure S1. Selected amide regions of 1H NMR spectra of SHB in presence of Mg2+. These NMR spectra were recorded on a 600 MHz Bruker NMR spectrometer using a cryoprobe. Bottom (blue): 200 µm of SHB in 10 mM HEPES buffer (pH 7.4) recorded at 298 K. Top (red): 200 µm of SHB + 5 mM Mg2+ in 5 mM Tris-D11 (pH 7.4) recorded at 308 K. The observed changes in the spectra suggest the significant structural differences due to the binding of Mg2+ ions to SHB.

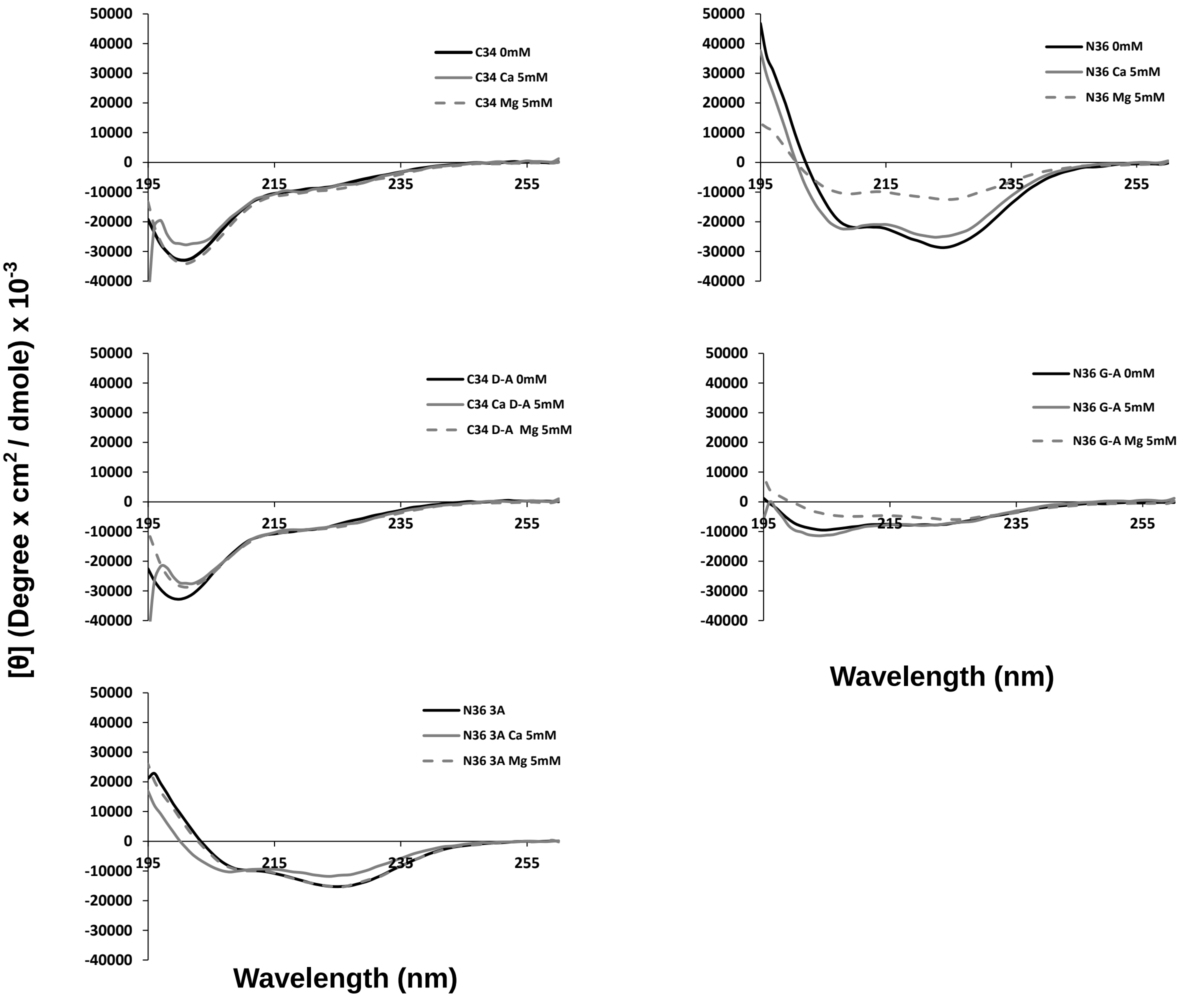

Figure S2. Ca2+ does not affect the overall secondary structure of WT and mutant C34 and N46 peptides. Peptides were measured at 25 µM in either PBS -/- (black) or PBS-/- supplemented with 5 mM of calcium (gray) or magnesium (dashed gray) ions.

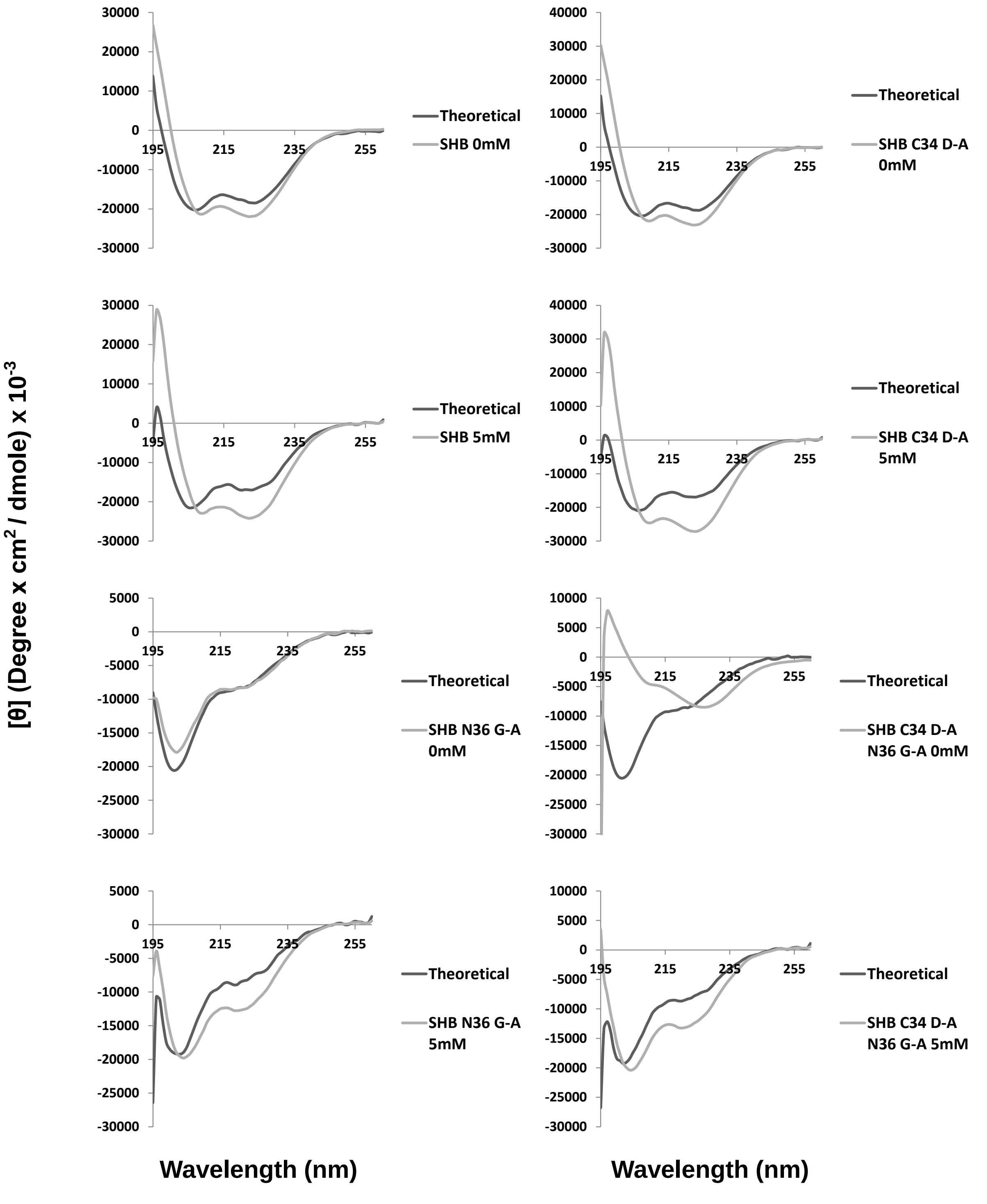

Figure S3. Theoretical and experimental spectra SHB variations tested in this study. Theoretical (black), experimental (gray). Peptides were measured at 25 µM. All but one of the SHB variations show a shift in their experimental spectra when compared to the theoretical one. SHB comprised of N36 G-A and WT C34 without Ca^{2+} did not show a major shift, suggesting that they do not interact.

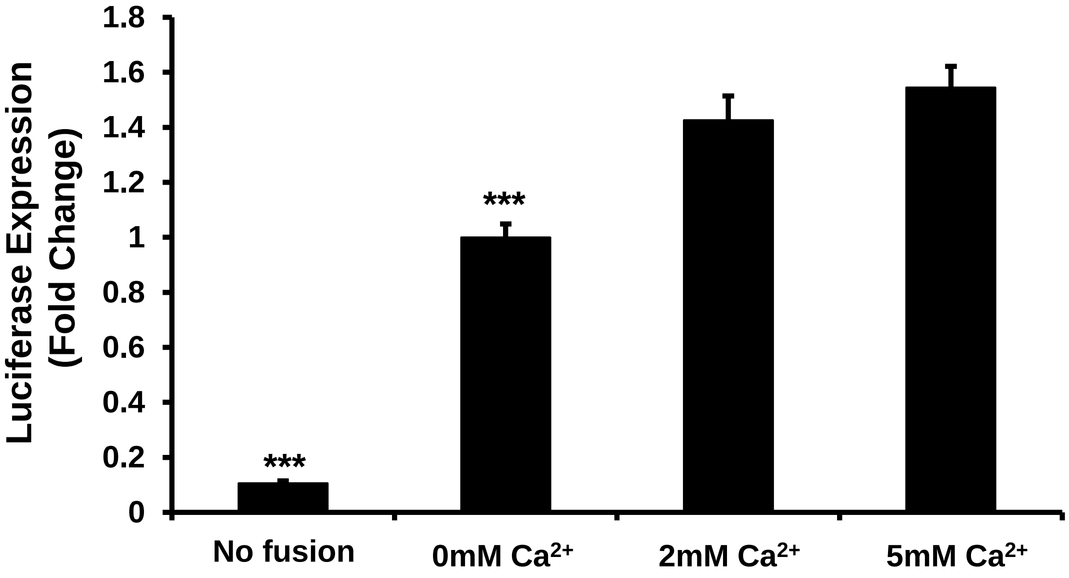

Figure S4: Calcium dependency of gp41 mediated cell-cell fusion. Cell-Cell fusion assay utilizing TZM-bl as target cells and HL2/3 as effector cells. Addition of 2 mM and 5 mM calcium ions significantly increased the rate of cell-cell fusion when compared to cells cultured in the absence of calcium ions. Luciferase expression was normalized to cells in media without calcium. n=3. *** P< 0.001. Error bars represent ± S.E.M.

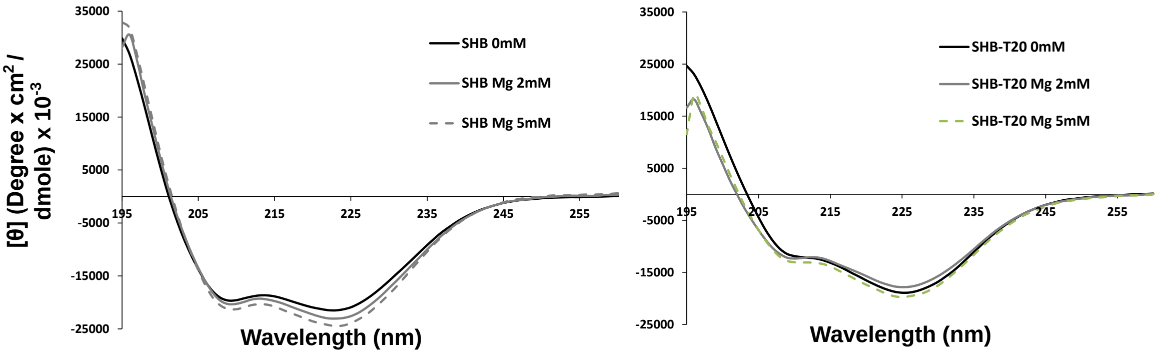

Figure S5. Mg^{2+} does not affect the structure C34 and T20 derived SHBs. Peptides were measured at 25 µM in either PBS -/- (Black) or PBS-/- supplemented with either 2 mM (grey) or 5 mM (dashed grey) magnesium ions.
